# AI ethics for the everyday intensivist

**DOI:** 10.1016/j.ccrj.2025.100115

**Published:** 2025-06-26

**Authors:** Sing Chee Tan, Lucy Modra, Tamishta Hensman

**Affiliations:** a Northern Health, Australia; b Austin Health, Australia

**Keywords:** ICU, Ethics, Artifical Intelligence, AI

## Abstract

In Australian intensive care units (ICUs), Artificial Intelligence (AI) promises to enhance efficiency and improve patient outcomes. However, ethical concerns surrounding AI must be addressed before widespread adoption. We examine the ethical challenges of of AI using the framework of the four pillars of biomedical ethics—beneficence, nonmaleficence, autonomy, and justice, and discuss the need for a fifth pillar of explicability. We consider the risks of perpetuating inequities, privacy breaches, and unintended harms, particularly in disadvantaged populations such as First Nations people. We advocate for a national strategy for ICUs to guide the ethical implementation of AI, that aligns with existing National AI Frameworks. Our recommendations for implementation of safe and ethical AI in ICU include education, developing guidelines, and ensuring transparency in AI decision-making. A coordinated strategy is essential to balance AI’s benefits with the ethical responsibility to protect patients and healthcare providers in critical care settings.

*You are purchasing a new ultrasound machine for your ICU. You are shown a machine with a built in artificial intelligence that helps guide probe position, perform haemodynamic calculations, and generate diagnoses. You are given a publication indicating it to be very accurate. What else would you need to consider regarding whether this AI should be used?*

## 1 Introduction

The field of artificial intelligence (AI) has seen significant advances in the last decade, bringing novel or improved applications to support medical decisions, predict adverse outcomes and optimise operational aspects of healthcare.^1^ In this article, we use the term 'AI' to describe computational systems that can perform tasks traditionally requiring human intelligence. This includes machine learning (ML); deep learning, a subset of ML; and, more recently, large language models.

In contrast to traditional technologies like rule-based algorithms (which rely on simple yes/no criteria), modern AI can learn from large data sets with minimal human supervision, enabling accurate predictions from complex data. For example, mechanical ventilators have traditionally required regular servicing at fixed intervals; AI incorporating data from various sensors can instead predict when component failure will occur and pre-emptively identify maintenance tasks.^2^

When faced with the complexities of evaluating an AI for use in the intensive care unit (ICU), it is possible for the everyday clinician to be overwhelmed with technical discussions and detail. Indeed, a recent systematic review indicated that the vast majority of ICU AI research is focused on performance metrics,^3^ and less so on addressing the basic ethical imperatives that underpin good clinical decision making, such as ensuring clinical benefit. Recognising the imperative for robust scrutiny of AI solutions in healthcare, ethical AI has been flagged as a priority at various levels, such as the World Health Organization and the Australian government.^4^^,^^5^

We aim to examine the ethical considerations in AI in the ICU from a traditional (and familiar) biomedical ethics lens and highlight the need for a consensus approach to guide the safe and ethical adoption of AI into intensive care practice.

## 2 AI ethics for the everyday clinician: the four (plus one) ethical pillars

Much in the same way that ICU clinicians fall back to the time-tested Airway-Breathing-Circulation framework across multiple resuscitation scenarios, we propose that established bioethical frameworks can continue to serve as the foundational structure for guiding our discussions and decision-making around AI use, owing to their familiarity, simplicity, and adaptability.

Specifically, we centre our discussion around the four known principles of biomedical ethics—**beneficence, nonmaleficence, autonomy, and justice**—with the addition of a fifth principle, **explicability**, which considers the distinctive challenge AI presents in understanding how recommendations are derived. It supports clinicians in asking practical questions such as: *Is this AI intervention safe for this patient? Does it respect their privacy? Can I explain or override the recommendation?*

1. **Beneficence.** Whilst most published AI models often have a high degree of accuracy and precision, these models need to undergo the scrutiny as any other of clinical solution, including consideration of external validity and ultimately whether they provides benefit for ICU patient.^6^ Despite the large amount of research into AI models for ICU, only a fraction of them have gone beyond the initial model design and validation, to real-world evaluation.^3^ This is accentuated by commercial interests, which may drive vendors to deploy AI solutions into use before peer-reviewed, multicentred validation studies. For example, a machine learning algorithm for predicting sepsis was commercialised and implemented at several health services before robust external validation. It was subsequently found to perform poorly once applied in separate contexts, with a high false positive rate, potentially leading to unnecessary or nonbeneficial interventions such as antibiotics or blood cultures.^7^ Notably, AI solutions in chest X-ray interpretation and cardiac imaging have demonstrated value in multicentre validation studies,^8^^,^^9^ highlighting that with rigorous evaluation, responsible and effective AI implementation is achievable.
2. **Nonmaleficence.** AI models are trained to pursue a narrow predefined objective, but they may not necessarily pursue the *intended* objective that aligns with clinician and patient values, despite having an appropriate, patient-relevant outcome measure.^10^ For example, an AI algorithm built to identify patients requiring additional healthcare support was trained using healthcare costs data. This led to an unintended conflation of lower healthcare costs–potentially due to systemic barriers in accessing healthcare–with the actual healthcare needs of the patient. As such, the algorithm was more likely to identify white patients as needing additional healthcare compared to black patients, despite performing ‘as designed’.^11^ Similar models were proposed during the COVID-19 pandemic to support decisions around ICU resource allocation. However, a considerable proportion of these models were subsequently found to have biased training data or incorporate predictor variables without accounting for underlying socio-economic disparities that were modifiers of these predictors.^12^^,^^13^ These examples flag the importance of ensuring that the AI is trained towards an objective that is ethically appropriate, without entrenching pre-existing inequity in healthcare that was encoded in historical data.
3. **Justice.** AI is at risk of creating inequity for several reasons. Models may be built using datasets that contain underrepresented groups. For example, algorithms to interpret chest X-rays based on gender-imbalanced datasets perform poorly in practice when applied to the underrepresented genders, despite initial high accuracy during development and training.^14^ This is particularly concerning with clinical data used to train AI models for ICU, where gender imbalance occurs across a range of diagnostic categories.^15^
In addition, by virtue of being trained on datasets derived from routine clinical practice (e.g. Electronic Medical Record data), AI models indirectly capture human biases in practice. For example, the Sequential Organ Failure Assessment score, commonly used as a predictor for mortality in AI algorithms, was found to consistently overestimate the risk of mortality for Black patients. The persistence of this effect even after adjustment indicates historically structural biases in practice, which led to worse outcomes for Black patients.^16^ When then applied in the context of triage tools, this led to inappropriate classification of more Black patients as ‘lower priority’ due to an inaccurate assessment of their mortality risk, diverting critical care resources away from them and further perpetuating racial inequity.
4. **Autonomy.** A major challenge AI poses to clinician and patient autonomy relates to the capacity to exercise a right to privacy.^17^ Although most data for training AI systems are anonymised, the secondary use of data from routine clinical practice raises other forms of privacy concerns for both clinicians and patients. First, AI may undermine epistemic privilege, or the right to withhold personal preferences and beliefs.^18^ Algorithms may be able to infer such personal information through patterns within large datasets, effectively removing the right of patients or clinicians to withhold this information.^19^
Second, individuals do not typically have consent and control over the use of their data for training AI. For example, clinicians and patients do not have the choice to opt out from their data being captured on Electronic Medical Records, which are then used for secondary analysis and training of models. While secondary data use is not unique to AI and is common in epidemiological studies, AI often involves the use of larger, more complex datasets, may draw inferences beyond what was explicitly disclosed (particularly when using unsupervised approaches), and are sometimes developed outside traditional ethics governance, such as for commercialisation.
These issues are of particular concern amongst First Nations people, for whom data sovereignty is highly valued in order to ensure that their own data are used to promote the interests of their communities in a culturally appropriate manner.^20^ In addition, being a minority group, they may also be more identifiable in datasets.
A second major area of concern regards the need for human oversight and responsibility over decisions involving AI. Good clinical practice is rooted in a shared-decision making framework, where clinicians and patients (or families) partner to respect the expertise, rights, and values of all participants.^21^ Despite a concerning narrative that AI may replace doctors,^22^ physicians must remain actively involved, interpreting AI outputs through the lens of patient values and clinical context, ensuring that every decision ultimately upholds the individual's right to informed and independent choices. This human-in-the-loop approach is essential for ethical, patient-centred care in the ICU.
5. **Explicability: A Particular Challenge of AI**

The unique characteristics of AI raise the need to consider a 5th ethical principle of explicability, or the ability to interrogate and understand how a particular recommendation was derived.^23^ Technological systems have traditionally been open to interpretation, even if the underlying processes are complex. For example, the mathematical model to calculate pulse-pressure variation is defined and understandable in terms of inputs and how they are manipulated to derive the output. In contrast, AI systems that make use of methods such as deep learning may be highly opaque in their ability to explain their outcomes. Unlike traditional models, which use interpretable variables (e.g. logistic regression), deep learning systems operate through multiple layers of abstraction, recognising patterns across thousands of data points which cannot be easily explained.

In the absence of explicability, AI systems cannot be assessed against any of the other pillars of ethics. For example, to assess whether an algorithm-driven treatment recommendation is likely to benefit an individual patient, we would need to assess whether that patient demographic is adequately represented in AI's dataset and whether the model output aligns with the patient's own values. This is of particular importance given the scale of AI implementations, the opacity of their inner workings, and the growing influence AI may have on decision-making.
The challenge of implementing explicability further lies in the difficult trade-off between transparency and accuracy. More complex computational methods such as deep learning may provide greater accuracy but are not explainable. Whilst the former is needed for physicians to understand and therefore critique how these recommendations were derived,^24^ patients may prefer a more accurate recommendation even if the factors underlying this recommendation remain opaque to them.^25^ Notably, the mechanisms underlying traditional medical recommendations may remain opaque to many, if not most, patients.

## 3 Moving beyond the basics

Just as the advanced practitioner may find the airway-breathing-circulation framework to be inadequate with increasing patient complexity, the four (plus one) pillars of biomedical ethics may be lacking with in more complex contexts, such as institutional, commercial, or societal settings.

Consider the procurement of a proprietary ICU decision support system: questions may arise around commercial incentives, who owns the data generated, and who is accountable if the AI underperforms. Stakeholders may need to draw upon a broader set of ethical paradigms when engaging with AI at a managerial, executive, or system level. These may include.

• **Utility and fairness**: Maximising overall benefit while ensuring equitable distribution of resources.
• **Fidelity and responsibility**: Upholding institutional trust, especially in commercial partnerships.
• **Pragmatism vs. idealism**: Balancing the urgency of implementation with the responsibility to have a highly effective solution.

Currently, there are no dedicated ethical frameworks to guide ICU leaders through the ethical complexities of AI procurement, implementation, and oversight from an organisational perspective. While principles such as fairness, accountability, and data stewardship are often referenced, these lack specificity for healthcare leadership. We propose that the development of an AI framework also be tailored to address the multilayered decisions faced by health systems integrating AI in different contexts.

*After thorough investigation, you identify several issues with the echocardiogram AI*.^26^*The AI has only been trained on cardiology patients in general wards, with the significant gender imbalance that is common within that diagnostic category. You also discover that images captured are sent to the manufacturer for training the next generation of their AI, which will be made available at significant cost. In addition, despite the high accuracy in validation studies, the AI provides no insight into how a diagnosis is derived*.^27^

## 4 Towards safe and ethical AI in ICUs

Many of the ethical challenges raised are not peculiar to ICU practice in Australia, and the ICU community would benefit from the development of a strategy to supporting ICU clinicians in the ethical implementation of AI, with capacity for a degree of contextualisation to the Australian regulatory environment. Promoting the ethical application of AI in ICU could involve:

Firstly, develop **training and education resources** to help clinicians understand and manage the ethical risks posed by AI. Medical school curricula often do not explicitly address the topic of artificial intelligence in the healthcare context,^28^ and it is not explicitly addressed in the ICU training programs such as the College of Intensive Care Medicine.^29^ Clinicians are left bereft of the skills and knowledge to evaluate and safely implement these technologies,^30^ highlighting the need for resources tailored specifically for the challenges of the ICU environment.^2^^,^^22^

Secondly, provide **frameworks and guidelines** for ethical evaluation of AI in ICU, similar to guidelines for other ICU technologies such as virtual care. There already exist a number of industry-agnostic resources, such as the National AI Ethics Principles, which could be contextualised to the ICU environment. As previously identified, there is also a need for frameworks to support ethical AI implementation from an ICU leadership perspective.

Thirdly, implement **governance mechanisms** around the use of AI in ICUs. This may occur at the professional level, such as including regulatory requirements to support learning about AI and ethics, and accreditation for Continuing Professional Development requirements. This would be in alignment with other Colleges, such as the Royal Australian College of Physicians, which now accredit digital health courses as acceptable for continuing professional development.

These mechanisms should also be implemented at the organisational level, and may include embedding the reporting of AI tools to existing safety and quality improvement committees and ensuring procurement processes are redesigned to enable robust evaluation of the AI technology.

Fourthly, ICU AI research needs to promote a **culture of interrogating the integrity of AI algorithms** being developed. This includes integration of guidelines such as Consolidated Standards of Reporting Trials-Artificial Intelligence (CONSORT-AI) and Standard Protocol Items: Recommendations for Interventional Trials-Artificial Intelligence (SPIRIT-AI) as a standard and setting expectations for AI trial reporting and operations. Practices such as keeping code open-source also promote a culture of openness and research scrutiny.

Recognising the complexity of advancing the safe and ethical use of AI in ICU, we also advocate for the establishment of a working group with a diverse representation that drives engagement with stakeholders (including patients, ethicists, and the wider ICU healthcare team), with reference to currently available resources and frameworks.

## 5 Conclusion

AI has great potential to improve patient care in ICU, but these opportunities come with ethical challenges that require thoughtful consideration. To date, many AI tools have underperformed when translated into real-world ICU settings, often due to biased training data, lack of external validation, or poor alignment with clinical goals. However, this should not overshadow the fact that some AI models have shown promise when developed and evaluated responsibly. The ethical implementation of AI in the ICU will require investment but will ultimately safeguard patients and clinicians while harnessing the benefits of this technology.

## Conflict of interest

The authors declare the following financial interests/personal relationships which may be considered as potential competing interests: Sing Chee Tan has received grant funding from the University of Melbourne for research in AI in healthcare.
